# Household

**Published:** 1889-11

**Authors:** 


### HOUSEHOLD.

### HOW TO USE A THERMOMETER.

In order to insure the proper reading of a thermometer, other things being equal, the instrument should be hung on the north side of a building and in the shade, being also suspended freely, that is, set out not less than four inches from the supporting substance of body, this distance being far enough out for the purpose, the readings of the instrument being thus made free from any interfering influence ; the best thermometers have an iron framework for this latter object, which can be fastened to the wall. On exposing a number of thermometers on different supporting materials, such as iron, tin, wood and stone, each one will be found to record a different temperature, quite marked ; the reason for this being, of course, the different conducting qualities of the substances. A brick wall, for example, becomes heated slowly but retains the heat for some time ; so, at certain times of the day, a thermometer hung on a brick wall will register the temperature inadequately in summer, while in winter it will register too high ; the reverse is nearly the case with iron, which radiates the heat quickly. The reflection or radiation of heat or light from neighboring objects is another disadvantage ; when, therefore, there is a tin roof or a white wall near by, the thermometer should be covered.

### RECEIPES.

*Ham Croquettes.*—One cup of ham, two cups of potatoes, one cup of bread crumbs, one tablespoonful of butter, and one egg. Make in balls, roll them in bread crumbs, and fry in hot fat.

*Omelet.*—Four eggs, yolks and whites beaten separately, and one and one-half tablespoonfuls of milk. Mix the whites and yolks together, and add a little salt and pepper, and turn into a buttered spider.

*Fish Moolie.*—Take some fried fish, two tablespoonfulls of cream, a dessert-spoonful of butter, three or four onions, green chillies (when they are to be had), a piece of ginger, and two or three tablespoonfuls of vinegar, boil for ten minutes, then serve. This is an excellent breakfast dish.

A SIMPLE TEST FOR FRESH EGGS.—This method is based upon the decrease in the density of eggs as they grow old. Dissolve two ounces of kitchen salt, says the account, in a pint of water, When a fresh laid egg is placed in this solution it will descend to the bottom of the vessel, while one that has been laid on the day previous will not quite reach the bottom. If the egg be three days old it will swim in the liquid ; if it is more than three days old it will float on the surface, and project above the latter more and more as they are older.

THE use of fatty matters as a dressing for the hair is very common and dates from a remote antiquity ; but it is a custom better honored in the breach than in the observance. Their use is uncleanly in the highest degree ; and serves no good purpose whatever. Bear's grease has long had a great reputation as a dressing for the hair ; but it does not differ from any other coarse animal fat, and possesses no particular beneficial properties. If a dressing of this sort must be used, a solution of castor-oil in cologne water is perhaps the best, or vaseline is a most excellent unguent which never turns rancid ; but the occasions are very few where it is a necessity.

